# Effect of Support Spacing on the Region-Specific Dimensional Accuracy of 3D-Printed Dental Models †

**DOI:** 10.3390/ma19163463

**Published:** 2026-08-15

**Authors:** Berker Alpöz, Burçin Akan, Ender Akan

**Affiliations:** 1 Department of Prosthodontics, Faculty of Dentistry, Izmir Katip Celebi University, 35620 Izmir, Türkiye; ender.akan@ikcu.edu.tr; 2 Department of Orthodontics, Faculty of Dentistry, Izmir Katip Celebi University, 35620 Izmir, Türkiye; burcin.akan@ikcu.edu.tr

**Keywords:** additive manufacturing, dental models, support spacing, masked stereolithography, dimensional accuracy, root mean square, digital dentistry, photopolymer resin

## Abstract

**Highlights:**

- Support spacing had a region-dependent effect on the dimensional accuracy of MSLA-printed dental models.
- Significant differences were detected in the global, marginal, and intaglio regions, but not in adjacent surfaces.
- The 3-mm configuration generally showed the most favorable dimensional performance.
- Global and intaglio analyses showed significant differences between the 3-mm and 7-mm groups.
- Region-specific RMS analysis may improve evaluation and optimization of support design.

**Abstract:**

Additive manufacturing is increasingly integrated into digital prosthodontic workflows for the fabrication of dental models; however, printing-related parameters may influence the dimensional accuracy of clinically relevant regions. This in vitro study evaluated the effect of support spacing on the region-specific dimensional accuracy of masked stereolithography (MSLA)-printed dental models. A standardized typodont model representing a three-unit posterior fixed dental prosthesis preparation in the FDI 24–26 region was digitized to obtain a reference STL dataset. Three support spacing configurations were evaluated: 3 mm, 5 mm, and 7 mm (*n* = 8 per group). All specimens were fabricated using an MSLA printer and a photopolymer model resin at a layer thickness of 50 μm. After post-processing, the printed models were rescanned and compared with the reference dataset using three-dimensional deviation analysis. Root mean square (RMS) deviations were calculated for global, marginal, intaglio, and adjacent regions. Significant differences were observed in the global (*p* < 0.001), marginal (*p* = 0.001), and intaglio (*p* = 0.010) regions, whereas adjacent regions were not significantly affected (*p* = 0.070). Increasing support spacing was associated with greater dimensional deviations in the global and intaglio regions. Overall, the 3-mm support spacing demonstrated the most consistent dimensional performance across the evaluated regions. These findings indicate that support spacing is an important design parameter influencing the dimensional predictability of MSLA-printed dental models and highlight the value of region-specific accuracy assessment for optimizing support design in digital prosthodontic workflows.

## 1. Introduction

Additive manufacturing (AM) technologies have transformed digital workflows in dentistry by enabling the rapid fabrication of patient-specific models, surgical guides, provisional restorations, and various laboratory components [1,2]. Among the available AM technologies, vat photopolymerization techniques have gained widespread acceptance because of their high dimensional accuracy, excellent surface quality, and ability to reproduce complex anatomical details [3]. Masked stereolithography (MSLA), a relatively recent development within vat photopolymerization systems, utilizes a liquid crystal display (LCD) mask to selectively polymerize photoreactive resin layers using ultraviolet light [4,5,6]. Compared with conventional stereolithography systems, MSLA offers faster production rates and reduced manufacturing costs while maintaining a high level of precision, making it particularly attractive for dental applications [7].

Despite these advantages, dimensional inaccuracies remain a significant concern in the fabrication of dental models and prosthetic components [8]. The accuracy of printed objects is influenced by numerous factors, including printer resolution, layer thickness, build orientation, post-processing procedures, resin composition, and support structure design [9,10]. In dental applications, even minor dimensional deviations may compromise the fit of restorations, affect occlusal relationships, and influence the accuracy of subsequent laboratory procedures [11,12]. Therefore, understanding the factors that contribute to dimensional distortion is essential for improving the reliability of additive manufacturing workflows.

During MSLA printing, dimensional changes may occur as a result of polymerization shrinkage, residual stress accumulation, and mechanical forces generated throughout the printing process. Each newly cured layer undergoes volumetric contraction during polymerization, while repeated layer-by-layer curing may lead to the development of internal stresses within the printed structure [13]. In addition, the separation of each cured layer from the release film generates peel forces that can induce deformation, particularly in unsupported or mechanically vulnerable regions [14]. The magnitude and distribution of these stresses are influenced not only by the material properties of the resin but also by the design and configuration of the support structures used during fabrication.

Support structures play a critical role in stabilizing printed objects throughout the manufacturing process. They provide mechanical reinforcement, reduce deformation during layer separation, and help maintain the intended geometry of the object until printing is completed. Several characteristics of support structures, including support density, geometry, placement strategy, contact point design, and support diameter, have been investigated in relation to printing accuracy [15].

Previous studies in the field of engineering have demonstrated that support design can significantly affect the dimensional stability of printed objects [15,16,17,18]. However, while support density and support configuration have received considerable attention, the specific influence of support spacing or density has been comparatively underexplored.

Support spacing may represent an important but overlooked parameter because it directly determines the distance between adjacent support contact points and consequently affects the length of unsupported spans within a printed structure [16]. As support spacing increases, larger unsupported regions may develop, potentially reducing local structural stiffness and increasing susceptibility to deformation caused by polymerization shrinkage and peel forces. Conversely, excessively dense support placement may increase material consumption, printing time, and post-processing requirements without necessarily improving accuracy. Therefore, identifying an optimal support spacing may contribute to a more efficient balance between dimensional accuracy and manufacturing efficiency.

Most previous investigations evaluating printing accuracy have relied primarily on global deviation measurements [19,20]. Although global analyses provide valuable information regarding overall dimensional trueness, they may not adequately capture localized distortions occurring in clinically important areas. Dental models often exhibit region-specific variations in accuracy because different anatomical regions are exposed to different stress distributions during printing and post-processing [21]. Consequently, evaluation of distinct regions may provide a more comprehensive understanding of how support parameters influence dimensional behavior. Assessing dimensional deviations separately in marginal, intaglio, adjacent, and global regions may reveal localized deformation patterns that remain undetected when only overall measurements are considered.

Although previous studies have investigated the effects of build orientation, layer thickness, and support density on the dimensional accuracy of printed dental models, limited evidence is available regarding the influence of support spacing and its region-specific effects [9,15,22]. Furthermore, the relationship between support spacing and localized dimensional deviations has not been sufficiently clarified in MSLA-printed dental models.

Therefore, the aim of this study was to evaluate the effect of different support spacing distances (3, 5, and 7 mm) on the dimensional accuracy of MSLA-printed dental models using three-dimensional deviation analysis. Accuracy was assessed globally and within predefined anatomical regions to identify potential region-specific deformation patterns associated with support spacing. The null hypothesis was that different support spacing distances would not significantly influence the dimensional accuracy of the printed models, either globally or within the evaluated regions.

## 2. Materials and Methods

### 2.1. Study Design

This in vitro study evaluated the effect of support spacing on the region-specific dimensional accuracy of MSLA-printed dental models. Three experimental groups were established according to the distance between adjacent support contact points: 3 mm, 5 mm, and 7 mm. Eight specimens were fabricated for each group (*n* = 8), resulting in a total of 24 printed models. All specimens were produced using the same printer, resin, layer thickness, build orientation, and post-processing protocol to isolate the effect of support spacing.

The sample size was determined by an a priori power analysis, as detailed in Section 2.6. The order of specimen fabrication and digitization was randomized using a computer-generated randomization sequence to minimize potential systematic bias.

Because eight specimens were included in each run, the three groups were distributed across the build platform in a 3-3-2 arrangement, and this allocation was rotated among the groups across the three print runs. Thus, each group contributed a total of eight specimens, while platform position and unequal within-run allocation were balanced across runs.

**Figure 1:**
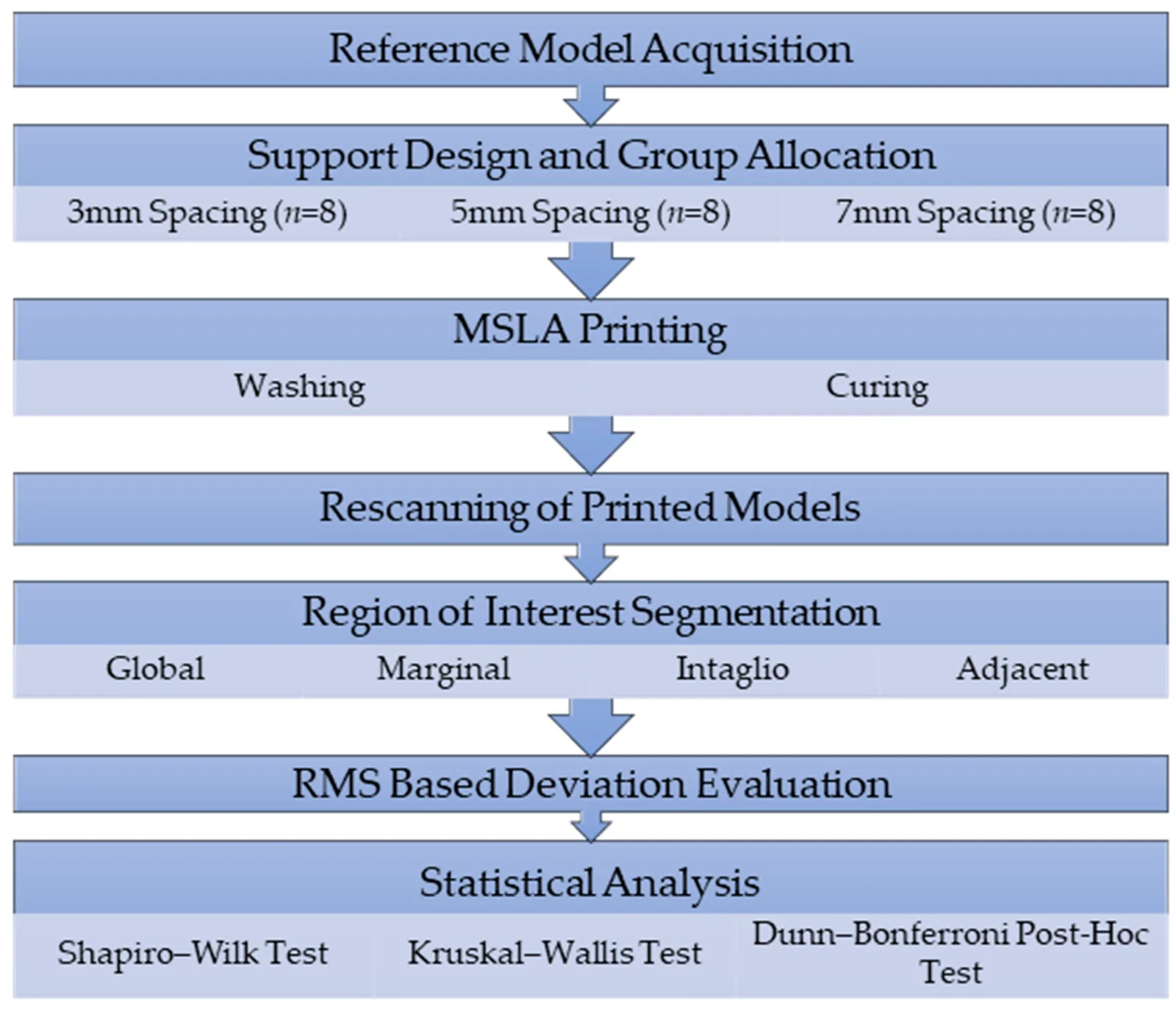
Overall workflow of the study.

The experimental workflow consisted of reference model acquisition, support spacing design, additive manufacturing, post-processing, digitization of printed specimens, alignment with the reference dataset, and region-based three-dimensional deviation analysis. The overall workflow is presented in Figure 1.

### 2.2. Reference Model Acquisition

A standardized typodont model (Frasaco AG-3, Frasaco GmbH, Tettnang, Germany) representing a three-unit posterior fixed dental prosthesis preparation in the FDI 24–26 region was used as the reference model. The model was digitized using a laboratory scanner (inEos X5, Dentsply Sirona, Bensheim, Germany) to obtain a high-resolution standard tessellation language (STL) dataset. 

This STL dataset was subsequently processed in the model creation module of AccuWare software version 3.2.1.109; Shining 3D, Hangzhou, China) to generate the final printable CAD model.

**Figure 2:**
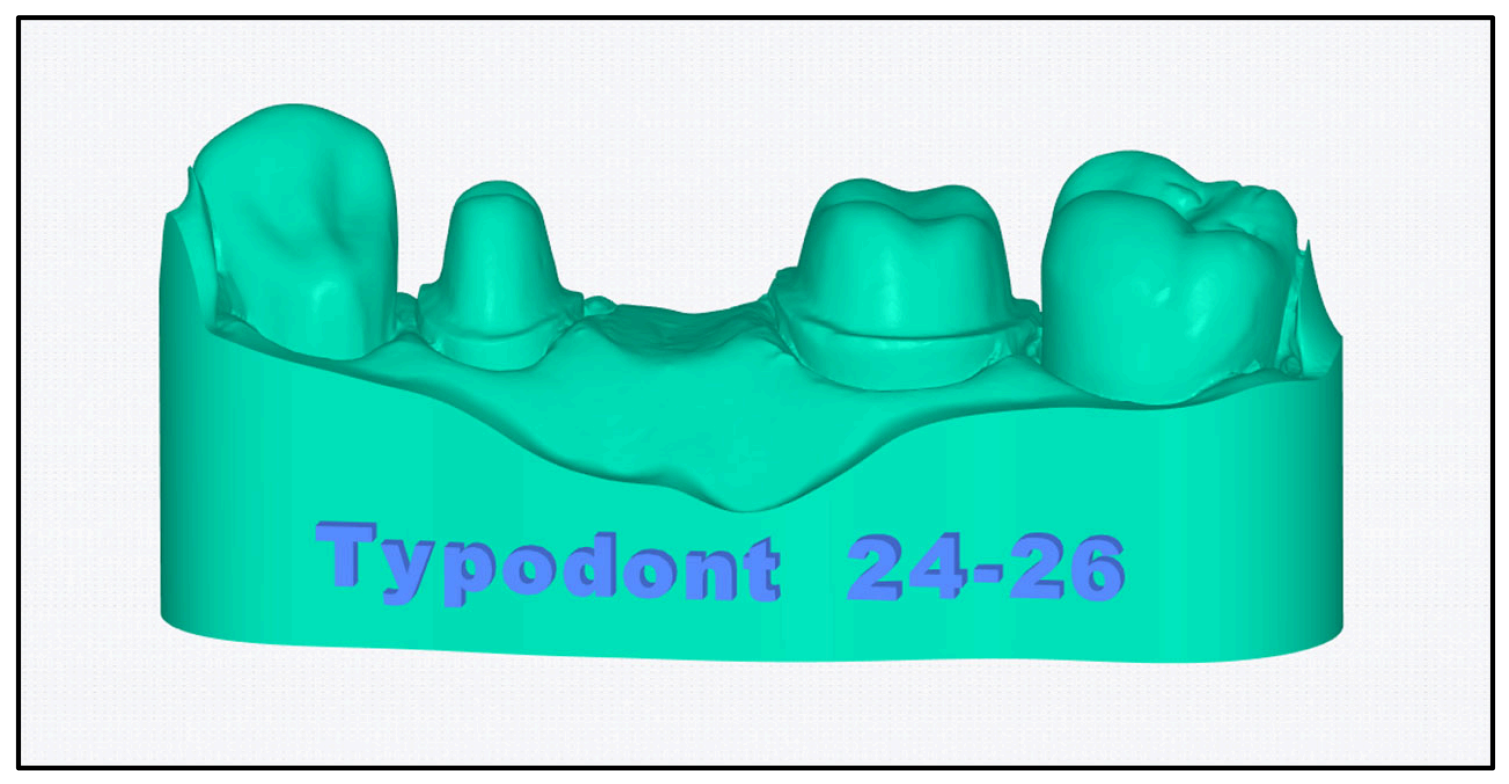
Three-dimensional view of the reference printable dental model.

Acquired scan data were imported into the model creation module of AccuWare software and converted into a printable dental model. In this step, the STL file corresponding to the FDI 24–26 region was processed to generate a standardized model geometry suitable for subsequent support generation and additive manufacturing. A 3D model view of this model-creation step is shown in Figure 2.

The scanned STL dataset was imported into the model creation module of AccuWare software and processed to generate a standardized printable dental model corresponding to the FDI 24–26 region. Trimming and boxing were performed to remove irrelevant scan extensions and establish the final printable geometry. The resulting printable CAD model, before support generation, was used as the reference dataset for all subsequent three-dimensional deviation analyses. Support structures were added to this otherwise unchanged geometry for fabrication, and the rescanned printed specimens were compared with the same printable CAD model.

Using the printable CAD model as the reference eliminated irrelevant scan extensions and ensured that the comparison was performed between geometrically identical models, differing only by the manufacturing process rather than by preprocessing geometry.

### 2.3. Support Spacing Design and Additive Manufacturing

The reference STL dataset was imported into AccuWare software (version 3.2.1.109; Shining 3D, Hangzhou, China) for support generation and print preparation Support spacing (3, 5, or 7 mm) served as the experimental variable. All remaining support generation parameters were kept constant throughout the study. The complete support generation settings are presented in Table 1.

Three support spacing configurations were created by modifying the center-to-center distance between adjacent support contact points, which was standardized as 3 mm, 5 mm, or 7 mm according to the assigned experimental group.

Support tip diameter, support shaft diameter, support penetration depth, support angle, and all other support-generation parameters were kept constant throughout the study. Only the distance between adjacent support contact points was modified.

**Figure 3:**
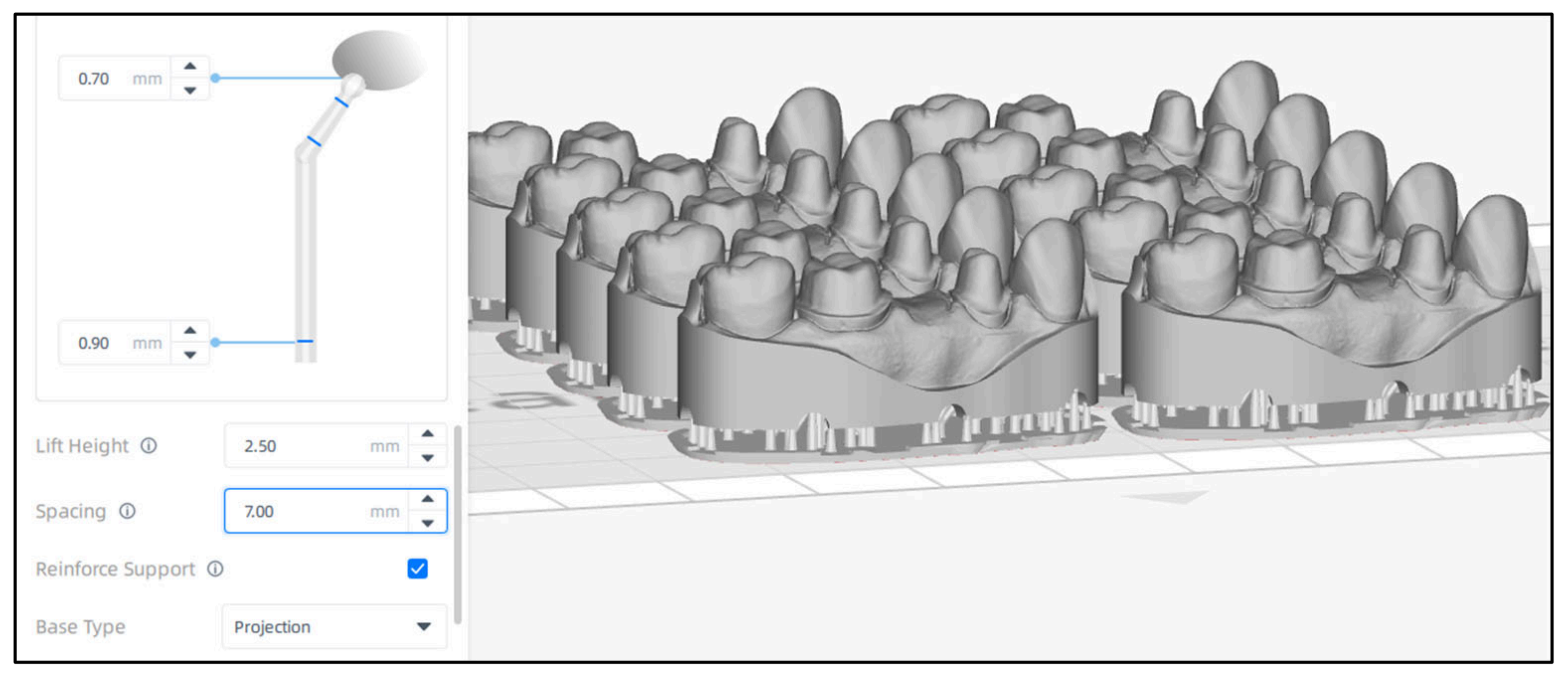
Support spacing configuration in the slicing software.

Except for support spacing, all support generation parameters and printing settings were kept constant across the experimental groups. A representative screenshot of the support spacing configuration in the slicing software is shown in Figure 3.

All specimens were fabricated using an MSLA printer (AccuFab-L4D, Shining 3D, Hangzhou, China) and a photopolymer model resin (M-PRINT Model Sand, Merz Dental GmbH, Lütjenburg, Germany). The resin was used with the validated material profile recommended for the AccuFab-L4D printing system. All models were printed using the same build orientation (0° relative to the build platform). A layer thickness of 50 μm was used for all specimens.

### 2.4. Post-Processing and Digitization of Printed Specimens

After printing, the specimens were removed from the build platform and cleaned twice in 96% isopropyl alcohol for 3 min each using an ultrasonic cleaning unit. An initial post-curing cycle was then performed using an Otoflash G171 unit (NK-Optik GmbH, Baierbrunn, Germany) according to the manufacturer’s validated protocol. The support structures were subsequently removed, followed by a second post-curing cycle to ensure complete polymerization of the exposed surfaces.

Each post-curing cycle consisted of 2400 xenon light flashes. The specimens were repositioned before the second cycle to ensure homogeneous polymerization of all exposed surfaces. The same cleaning, post-curing, support removal, and final post-curing procedures were applied to all specimens.

**Figure 4:**
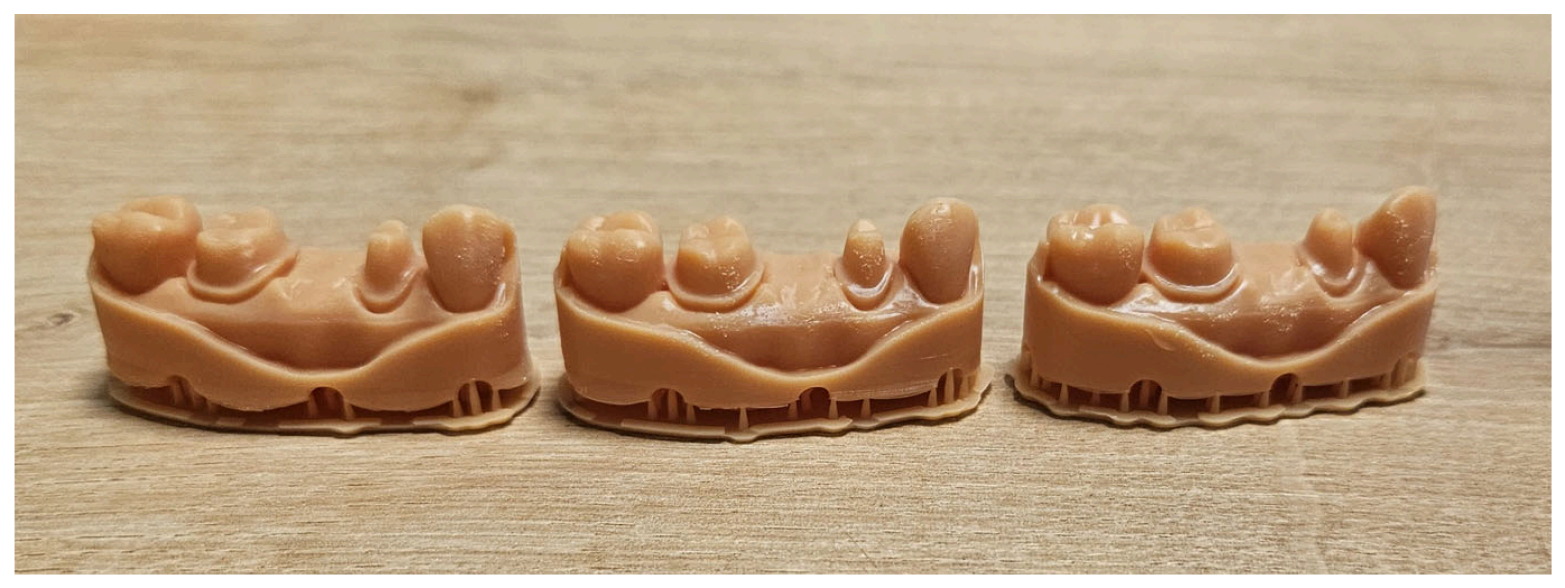
Representative printed specimens from the 3-mm, 5-mm, and 7-mm support-spacing groups. Differences in support density are visually apparent among the three configurations.

**Figure 5:**
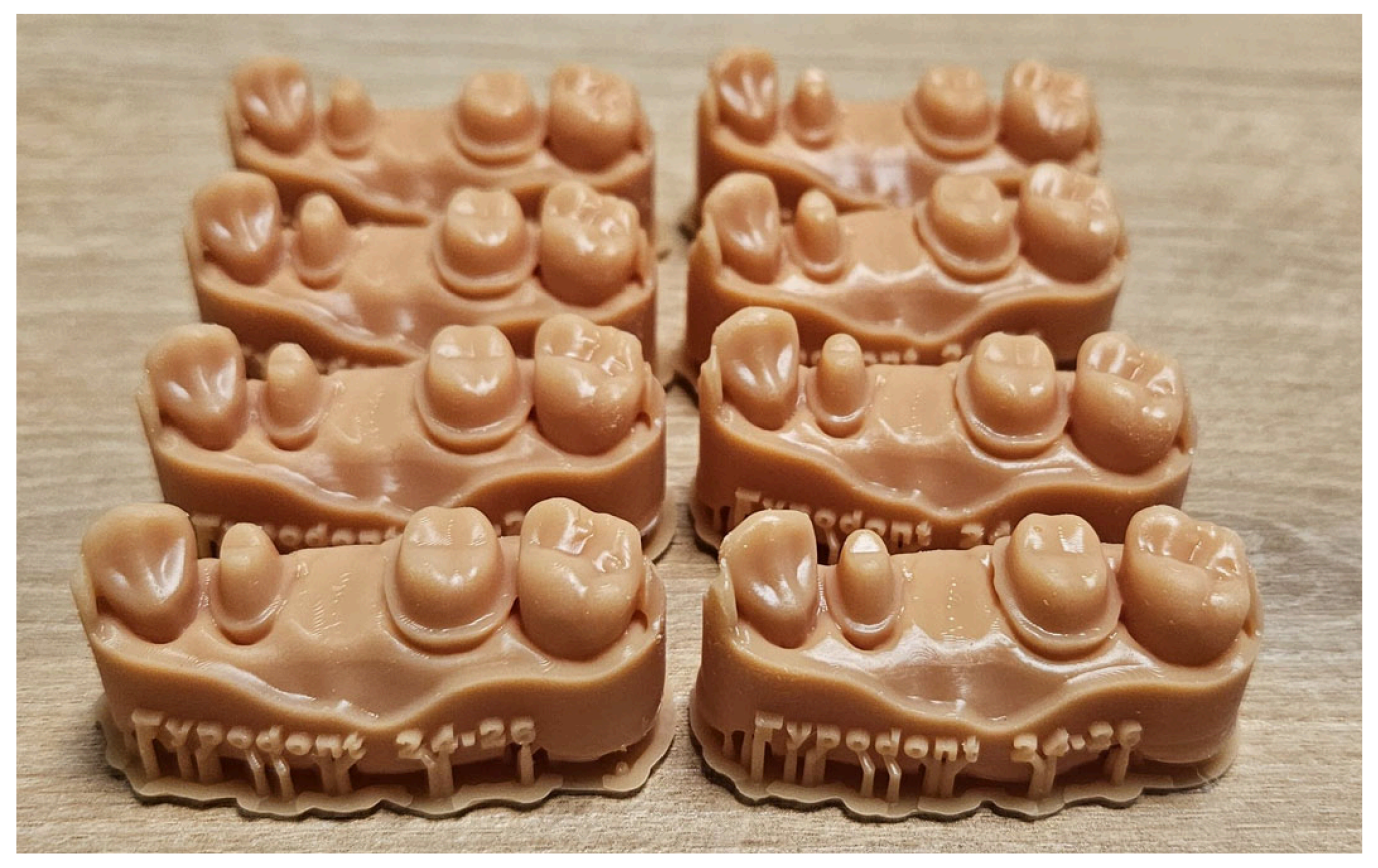
Representative arrangement of eight specimens produced within a single print session.

Each printed specimen was rescanned using the same laboratory scanner used for reference model acquisition. The resulting STL datasets were exported for three-dimensional deviation analysis. Representative printed specimens from the three support-spacing groups and the arrangement of eight specimens within a single print session are shown in Figure 4 and Figure 5, respectively.

### 2.5. Three-Dimensional Deviation Analysis

Three-dimensional deviation analysis was performed using Geomagic Qualify 2012 software (3D Systems, Rock Hill, SC, USA). Each test STL dataset was initially aligned with the reference STL dataset, followed by global best-fit alignment using the automated registration function of the software. After alignment, dimensional discrepancies between the printed model and the reference dataset were quantified using root mean square (RMS) deviation values. RMS values were converted to micrometers for reporting.

Four regions of interest were evaluated separately: global, marginal, intaglio, and adjacent regions. Global best-fit alignment and RMS calculation were performed using all anatomical model surfaces coronal to the boxing boundary. The model base and support-contact areas were excluded from both alignment and deviation analysis. The marginal ROI comprised a standardized 0.5-mm-wide band extending coronally from the cervical finish line of both prepared abutments. The intaglio ROI included the complete prepared surfaces of the abutment teeth coronal to the marginal band, including the axial walls, internal line angles, and occlusal surfaces. 

**Figure 6:**
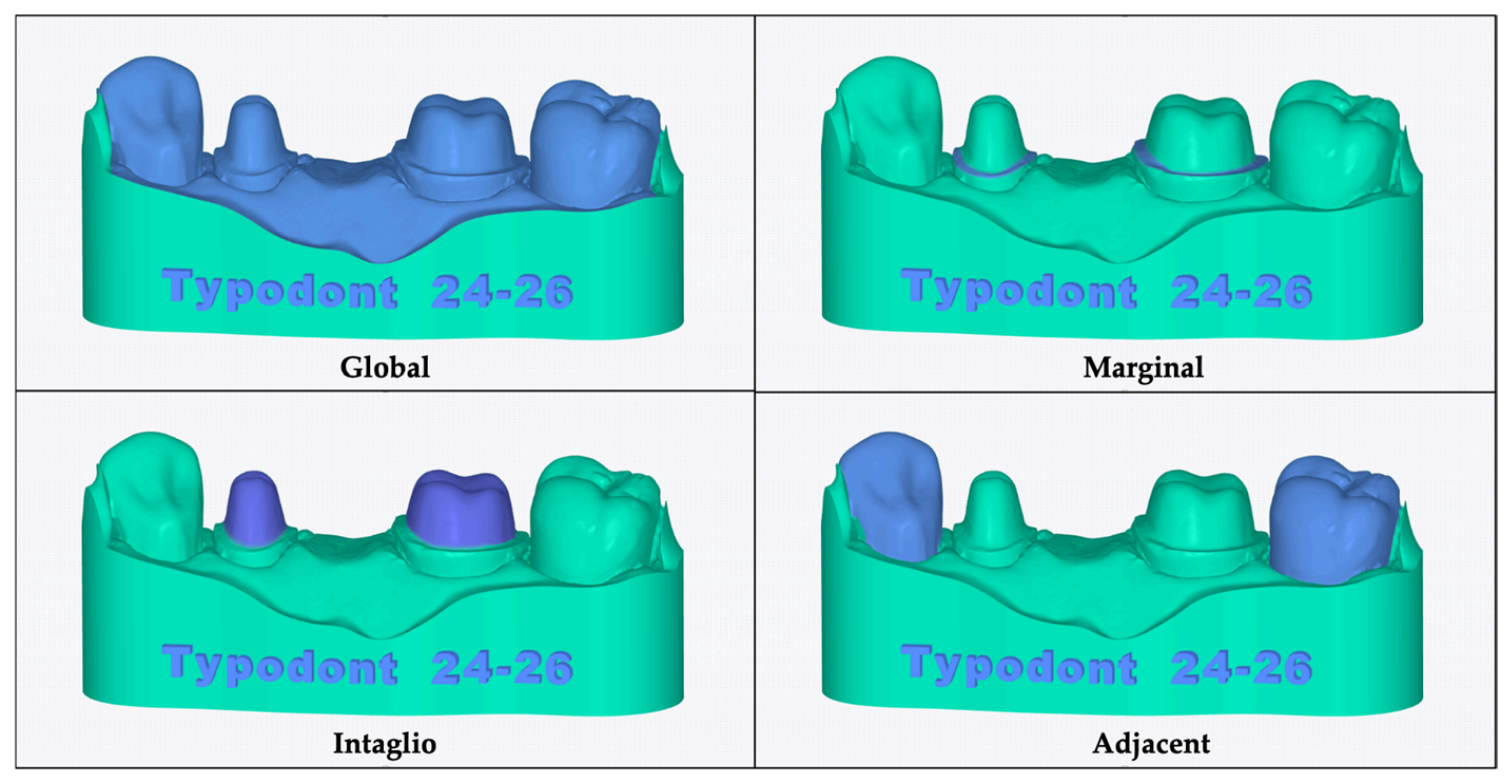
Visualization of the four regions of interest (ROIs) defined on the final printable reference CAD model. The highlighted surfaces represent the global, marginal, intaglio, and adjacent regions included in the respective regional deviation analyses, whereas the nonselected surfaces are shown in green.

The adjacent ROI comprised the external tooth surfaces neighboring the prepared abutments and excluded the prepared abutment surfaces, marginal band, model base, and support-contact areas. All ROI boundaries were defined once on the final printable reference CAD model and were subsequently transferred unchanged to each aligned test dataset. The four ROIs defined on the final printable reference CAD model are illustrated in Figure 6.

This standardized approach minimized examiner-dependent variability in ROI selection. To assess intraobserver reliability, eight STL datasets were randomly selected and reanalyzed by the same examiner after a two-week interval. Agreement between the initial and repeated measurements was evaluated using a two-way mixed-effects, single-measure intraclass correlation coefficient based on absolute agreement, with 95% confidence intervals.

### 2.6. Statistical Analysis

Statistical analyses were performed using SPSS software (version 26.0; IBM Corp., Armonk, NY, USA). Normality was assessed separately for each region using the Shapiro–Wilk test applied to the model residuals. The residuals showed significant deviations from normality in the marginal and intaglio regions. Considering these findings and the small group sizes, non-parametric statistical methods were applied consistently across all regions.

Intergroup comparisons were performed separately for each region of interest using the Kruskal–Wallis test. When a significant difference was detected, pairwise comparisons were conducted using Dunn–Bonferroni post-hoc tests. The level of statistical significance was set at α = 0.05.

An a priori power analysis was performed using G*Power software (version 3.1.9.6) for a one-way fixed-effects ANOVA with three groups. Based on an expected effect size of Cohen’s f = 0.72, derived from previously reported RMS deviation data [23], an alpha level of 0.05, and a target power of 0.80, the minimum required total sample size was calculated as 22 specimens. To ensure equal group allocation, eight specimens were included in each group, resulting in a total sample size of 24.

## 3. Results

Descriptive statistics for all regions and support-spacing groups are provided in Table A1 (Appendix A), while the results of the Kruskal–Wallis tests and Dunn–Bonferroni post-hoc comparisons are summarized in Table 2.

### 3.1. Global Analysis

Global RMS values increased as support spacing increased. The median RMS values were 243.0 µm, 295.8 µm, and 352.0 µm for the 3-mm, 5-mm, and 7-mm groups, respectively. The corresponding mean RMS values were 244.5 ± 33.2 µm, 295.0 ± 20.9 µm, and 359.1 ± 42.2 µm.

**Figure 7:**
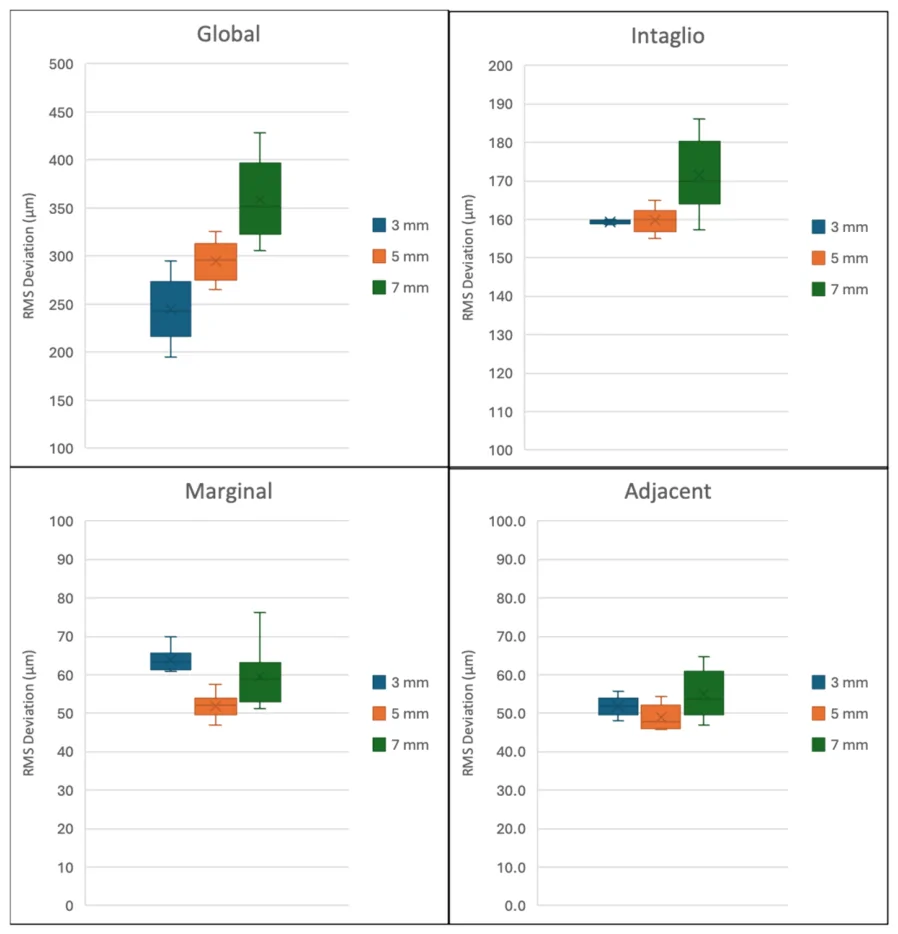
Box plots showing the distributions of RMS deviation values in the global, intaglio, marginal, and adjacent regions according to support spacing.

A statistically significant difference was detected among the groups in the global analysis (Kruskal–Wallis, *p* < 0.001). Dunn–Bonferroni post-hoc comparisons showed a significant difference between the 3-mm and 7-mm groups (adjusted *p* < 0.001). No significant differences were observed between the 3-mm and 5-mm groups (adjusted *p* = 0.143) or between the 5-mm and 7-mm groups (adjusted *p* = 0.085). The distributions of RMS deviation values across the three support-spacing groups for all evaluated regions are presented in Figure 7.

### 3.2. Marginal Analysis

For the marginal region, median RMS values were 63.3 µm, 52.0 µm, and 59.0 µm for the 3-mm, 5-mm, and 7-mm groups, respectively. Mean RMS values were 63.9 ± 3.0 µm, 52.0 ± 3.2 µm, and 59.6 ± 8.0 µm.

A statistically significant difference was identified among the groups in the marginal analysis (H = 14.105, *p* = 0.001). Post-hoc comparisons revealed a significant difference between the 3-mm and 5-mm groups (adjusted *p* = 0.001). The differences between the 3-mm and 7-mm groups (adjusted *p* = 0.290) and between the 5-mm and 7-mm groups (adjusted *p* = 0.111) were not statistically significant.

### 3.3. Intaglio Analysis

Among the region-specific ROIs, the intaglio region demonstrated higher RMS deviation values than the marginal and adjacent regions. The median RMS values were 159.5 µm, 160.1 µm, and 169.8 µm for the 3-mm, 5-mm, and 7-mm groups, respectively. Mean RMS values were 159.4 ± 0.4 µm, 159.8 ± 3.2 µm, and 171.4 ± 9.7 µm, respectively.

A statistically significant difference was observed among the groups in the intaglio analysis (H = 9.285, *p* = 0.010). Pairwise comparisons showed a significant difference between the 3-mm and 7-mm groups (adjusted *p* = 0.013). No significant difference was found between the 3-mm and 5-mm groups (adjusted *p* = 1.000). The difference between the 5-mm and 7-mm groups did not reach statistical significance after adjustment (adjusted *p* = 0.059).

### 3.4. Adjacent Analysis

Adjacent regions showed the lowest sensitivity to changes in support spacing. Median RMS values were 51.8 µm, 47.8 µm, and 53.6 µm for the 3-mm, 5-mm, and 7-mm groups, respectively. Mean RMS values were 51.8 ± 2.6 µm, 49.0 ± 3.3 µm, and 55.0 ± 6.2 µm.

No statistically significant difference was detected among the groups in the adjacent region (H = 5.314, *p* = 0.070). Therefore, no post-hoc pairwise comparisons were performed for this region. Intraobserver agreement for the global ROI was excellent, with an intraclass correlation coefficient of 0.993 (95% CI: 0.968–0.999).

Overall, support spacing affected dimensional accuracy in a region-dependent manner. Significant group effects were observed in the global, marginal, and intaglio regions, whereas adjacent surfaces were not significantly affected. These findings indicate that region-based analysis provides additional information beyond global RMS values alone.

## 4. Discussion

The present study evaluated the influence of support spacing on the region-specific dimensional accuracy of MSLA-printed dental models. The null hypothesis was partially rejected because support spacing significantly affected the global, marginal, and intaglio RMS deviation values, whereas no significant differences were observed for the adjacent region. These findings indicate that the influence of support spacing is region-dependent rather than uniform across the printed model. More importantly, the results demonstrate that evaluating clinically relevant anatomical regions separately provides additional information that may not be revealed by global deviation analysis alone.

The global analysis demonstrated a progressive increase in RMS deviation with increasing support spacing, with the 7-mm configuration exhibiting significantly greater deviations than the 3-mm configuration. This finding suggests that enlarging the unsupported distance between adjacent supports may reduce the overall dimensional stability of the printed model during fabrication. Support structures not only prevent print failure but also provide mechanical stabilization throughout layer-by-layer polymerization and separation from the release film.

As the unsupported span increases, the printed structure becomes more susceptible to elastic deformation, residual stress accumulation, and polymerization-induced distortion. Previous engineering studies have similarly reported that support-related parameters influence the dimensional stability of photopolymerized components by altering the mechanical resistance of unsupported regions during printing.

Although most previous dental investigations have focused on support density or build orientation rather than support spacing, the present findings extend current knowledge by demonstrating that the distance between adjacent support contacts itself represents an independent factor influencing dimensional accuracy. These findings are consistent with the observations of Hussein and Hussein [15], who demonstrated that support-related design parameters significantly influence the trueness of additively manufactured dental frameworks. However, unlike their study, which primarily evaluated support structure density, the present investigation specifically focused on the spacing between adjacent support contact points, providing additional evidence that support spacing itself represents an independent determinant of dimensional accuracy.

The observed relationship between support spacing and dimensional accuracy is biologically and mechanically plausible. During MSLA printing, each newly polymerized layer undergoes volumetric shrinkage while simultaneously being subjected to repeated peel forces generated during separation from the vat film. These cyclic mechanical forces may induce localized deformation, particularly in regions with insufficient structural reinforcement. When support spacing is increased, the effective unsupported span also increases, reducing local rigidity and permitting greater deflection during layer separation. Consequently, both polymerization shrinkage and peel-induced mechanical stresses may accumulate more readily, leading to greater dimensional discrepancies. These mechanisms have previously been described in vat photopolymerization systems and provide a reasonable explanation for the trend observed in the present study. A similar relationship between polymerization-induced stresses and dimensional distortion has been described in previous investigations evaluating vat photopolymerization technologies, supporting the hypothesis that both polymerization shrinkage and peel-induced mechanical loading contribute to cumulative dimensional deviations during layer-by-layer fabrication [13].

The intaglio surface showed a significant response to changes in support spacing. RMS deviations increased from approximately 159 μm in the 3-mm and 5-mm groups to approximately 171 μm in the 7-mm group, with a statistically significant difference identified between the 3-mm and 7-mm configurations. Although the absolute increase was modest, this finding may have clinical relevance, as dimensional deviations in the intaglio surface could affect the internal adaptation of indirect restorations. Unlike relatively flat external surfaces, the intaglio region consists of complex concave geometries, steep axial walls, internal line angles, and occlusal morphology. Such anatomical characteristics are inherently more susceptible to localized deformation caused by polymerization shrinkage and mechanical stresses during printing. Previous investigations evaluating the internal surfaces of additively manufactured restorations have likewise demonstrated that geometrically complex internal regions are generally more sensitive to manufacturing-related inaccuracies than external surfaces. Therefore, the present findings support the concept that optimizing support design may be particularly important for preserving dimensional fidelity in clinically critical internal areas.

These observations are in agreement with the studies of Metin et al. [24] and Revilla-León et al. [25], who reported that complex internal geometries exhibit greater manufacturing inaccuracies than external surfaces because of their anatomical complexity and limited accessibility during additive manufacturing. Similarly, Kaffaf et al. [26] demonstrated that internal surface accuracy remains one of the most critical determinants of restoration adaptation, further emphasizing the clinical relevance of preserving dimensional fidelity within the intaglio region.

The marginal region also exhibited statistically significant differences among the evaluated support spacing configurations. Interestingly, however, the significant pairwise difference occurred between the 3-mm and 5-mm groups rather than between the 3-mm and 7-mm groups. This non-linear response suggests that marginal accuracy is influenced by multiple interacting factors rather than support spacing alone. The finish-line area represents a relatively narrow anatomical structure where local support distribution, preparation geometry, alignment during best-fit registration, scanner resolution, and post-processing procedures may all contribute to measurement variability. Consequently, the absence of a progressive increase in marginal deviation with increasing support spacing should not necessarily be interpreted as contradictory to the global findings. Instead, it indicates that localized geometric characteristics may modify the influence of support spacing on specific anatomical regions. Similar variability has also been reported in previous studies evaluating marginal adaptation of additively manufactured restorations, emphasizing that marginal accuracy is determined by several manufacturing variables acting simultaneously. Previous studies evaluating marginal adaptation of digitally manufactured restorations have similarly reported that marginal accuracy is influenced by multiple manufacturing variables rather than a single printing parameter alone. Therefore, the absence of a linear response in the present study is not unexpected and likely reflects the multifactorial nature of marginal dimensional behavior [27,28,29,30].

In contrast, the adjacent region did not show statistically significant differences among the experimental groups. These surfaces represent relatively continuous external anatomical areas with greater structural continuity and fewer abrupt geometric transitions than preparation margins or internal surfaces. Such characteristics may render them less susceptible to localized deformation generated during polymerization and layer separation. The stability observed in the adjacent region further supports the concept that support spacing does not affect all anatomical regions equally and highlights the importance of region-specific evaluation rather than relying exclusively on global deviation measurements.

An important contribution of the present study is the use of region-based three-dimensional deviation analysis. Most previous investigations evaluating the dimensional accuracy of printed dental models have primarily reported global trueness values. Although these measurements provide valuable information regarding the overall dimensional behavior of printed models, they may obscure localized inaccuracies occurring in clinically important regions. In prosthodontic applications, deviations affecting the preparation margin or internal surface may have substantially greater clinical implications than comparable deviations occurring elsewhere on the model.

By separately evaluating global, marginal, intaglio, and adjacent regions, the present study provides a more comprehensive understanding of how support spacing influences dimensional behavior and demonstrates that different anatomical regions respond differently to identical manufacturing conditions. Similar conclusions have been reported by Ender and Mehl [19] and Etemad-Shahidi et al. [20], who emphasized that overall trueness measurements may not adequately reflect localized dimensional discrepancies occurring in clinically relevant areas. The present study further expands this concept by demonstrating that support spacing affects individual anatomical regions differently, reinforcing the value of region-specific accuracy assessment.

Although statistically significant differences were identified, their clinical significance should be interpreted cautiously. Statistical significance demonstrates that dimensional deviations differed between support spacing configurations under standardized experimental conditions; however, it does not necessarily indicate that these differences would adversely affect the clinical performance of restorations. The clinical relevance of dimensional discrepancies depends largely on the intended application of the printed model.

Diagnostic casts may tolerate greater deviations than models used for definitive prosthodontic restorations, where internal adaptation and marginal integrity directly influence restoration fit and long-term clinical success. Likewise, acceptable dimensional tolerances vary according to whether the printed model is intended for diagnosis, treatment planning, surgical guide fabrication, provisional restorations, or definitive prosthodontic procedures.

In the present study, the 3-mm support spacing generally demonstrated the most favorable dimensional performance. Nevertheless, these findings should not be interpreted as evidence that this configuration is universally optimal for every clinical situation. The present investigation evaluated dimensional accuracy only and did not assess restoration fit, marginal adaptation after fabrication, or clinical outcomes. Previous systematic reviews have also highlighted that clinically acceptable dimensional discrepancies remain application-dependent and that no universal threshold can be applied across all prosthodontic workflows [29,30,31].

Consequently, interpretation of RMS values should always consider the intended clinical application rather than statistical significance alone. Future investigations correlating regional RMS deviations with restoration fit and clinical adaptation would further clarify the practical implications of these findings.

From a manufacturing perspective, selecting a smaller support spacing also involves practical trade-offs. Increasing the number of support structures may enhance dimensional stability by reducing unsupported spans, but it may simultaneously increase resin consumption, printing time, support removal procedures, and post-processing complexity. These parameters were intentionally kept outside the scope of the present investigation and therefore were not quantified.

Consequently, the present findings should be interpreted as demonstrating the influence of support spacing on dimensional behavior rather than recommending a universal support configuration. The optimal spacing is likely to depend on multiple factors, including model geometry, resin properties, printing technology, support design strategy, and the intended clinical application. Similar trade-offs between printing accuracy, manufacturing efficiency, and material consumption have previously been discussed by Ravi and Chen [18], who demonstrated that optimization of support parameters requires balancing dimensional accuracy against printing time and material usage.

Several limitations should be acknowledged. Only one MSLA printer, one photopolymer resin, one layer thickness, one build orientation, and one support-generation software were evaluated. Therefore, the present findings cannot be directly generalized to other additive manufacturing systems, materials, or printing workflows. Furthermore, the study was performed under standardized in vitro conditions using a typodont model, which may not fully reproduce the anatomical variability encountered in clinical practice. Finally, the investigation focused exclusively on dimensional accuracy and did not evaluate the fit or clinical performance of restorations fabricated on the printed models.

Future studies should investigate the combined influence of support spacing with other printing-related parameters, including support geometry, support density, build orientation, layer thickness, resin composition, and post-curing protocols. In addition, correlating regional dimensional deviations with restoration fit, marginal adaptation, occlusal accuracy, and manufacturing efficiency would facilitate the development of evidence-based guidelines for optimizing support design in digital prosthodontic workflows. Similar research directions have recently been encouraged in systematic reviews evaluating additive manufacturing workflows, which emphasized the need to integrate dimensional accuracy with clinically relevant outcomes such as restoration fit, internal adaptation, and manufacturing efficiency [22].

## 5. Conclusions

Within the limitations of this in vitro study, support spacing significantly influenced the dimensional accuracy of MSLA-printed dental models in a region-dependent manner. Increasing the spacing between adjacent support structures resulted in significantly greater RMS deviations in the global and intaglio analyses, whereas the adjacent region remained unaffected. Although the marginal region also demonstrated statistically significant differences, its response pattern was not linear, suggesting that additional geometric and manufacturing factors may contribute to marginal dimensional behavior.

Among the evaluated configurations, the 3-mm support spacing generally demonstrated the most favorable dimensional performance, particularly in the global and intaglio regions. Nevertheless, these findings should be interpreted as evidence of improved dimensional predictability rather than as a universal recommendation for all printing workflows. The optimal support spacing is likely to depend on multiple variables, including model geometry, resin characteristics, printer technology, support design strategy, and the intended clinical application.

The present findings highlight the importance of region-specific three-dimensional deviation analysis, which provides additional information beyond conventional global accuracy measurements and may facilitate optimization of support design for digitally manufactured dental models. However, because only a single MSLA printer, one photopolymer resin, one build orientation, and one support-generation software were evaluated, further studies involving different manufacturing systems and direct assessments of restoration fit are required before these findings can be generalized to broader clinical applications. Future investigations correlating regional RMS deviations with restoration fit and manufacturing efficiency will help establish evidence-based guidelines for support design optimization in digital prosthodontics.

## Figures and Tables

**Table 1:** Support generation parameters used for all experimental groups.

Parameter	Setting
Support spacing (experimental variable)	3, 5, or 7 mm
Support pillar height	2.50 mm
Maximum support face angle	50°
Edge offset	0.20 mm
General contact ball diameter	0.70 mm
Insert depth ratio	0.75
Middle-part diameter	0.90 mm
Inner support	Enabled
Reinforce support	Enabled
Base type and height	Projection; 0.80 mm

**Table 2:** Kruskal–Wallis and post-hoc pairwise comparisons.

Region	Kruskal–Wallis H	df	*p*-Value	Significant Pairwise Comparison
Global	17.420	2	<0.001	3 mm vs. 7 mm, adj. *p* < 0.001
Marginal	14.105	2	0.001	3 mm vs. 5 mm, adj. *p* = 0.001
Intaglio	9.285	2	0.010	3 mm vs. 7 mm, adj. *p* = 0.013
Adjacent	5.314	2	0.070	Not significant

## Data Availability

The original contributions presented in this study are included in the article. Further inquiries can be directed to the corresponding author.

## Abbreviations

The following abbreviations are used in this manuscript:

3D	Three-dimensional
AM	Additive manufacturing
CAD	Computer-aided design
FDI	Fédération Dentaire Internationale
ICC	Intraclass correlation coefficient
ICP	Iterative closest point
LCD	Liquid crystal display
MSLA	Masked stereolithography
ROI	Region of interest
RMS	Root mean square
SD	Standard deviation
SPSS	Statistical Package for the Social Sciences
STL	Standard tessellation language
UV	Ultraviolet

## Appendix A

**Table A1:** Descriptive statistics of RMS deviation values for each region.

Region	Support Spacing	*n*	Mean ± SD (µm)	Median (Q1–Q3) (µm)
Global	3 mm	8	244.5 ± 33.2	243.0 (225.5–264.4)
Global	5 mm	8	295.0 ± 20.9	295.8 (279.3–309.8)
Global	7 mm	8	359.1 ± 42.2	352.0 (328.9–386.8)
Marginal	3 mm	8	63.9 ± 3.0	63.3 (61.7–65.0)
Marginal	5 mm	8	52.0 ± 3.2	52.0 (50.4–53.4)
Marginal	7 mm	8	59.6 ± 8.0	59.0 (54.2–61.1)
Intaglio	3 mm	8	159.4 ± 0.4	159.5 (159.1–159.7)
Intaglio	5 mm	8	159.8 ± 3.2	160.1 (157.9–161.6)
Intaglio	7 mm	8	171.4 ± 9.7	169.8 (165.6–178.4)
Adjacent	3 mm	8	51.8 ± 2.6	51.8 (50.0–53.6)
Adjacent	5 mm	8	49.0 ± 3.3	47.8 (46.4–51.5)
Adjacent	7 mm	8	55.0 ± 6.2	53.6 (50.8–59.7)
